# Self‐Assembled Hydroxypropyl Celluloses With Structural Colors for Biomedical Applications

**DOI:** 10.1002/smmd.70004

**Published:** 2025-04-20

**Authors:** Zhuohao Zhang, Luoran Shang

**Affiliations:** ^1^ Shanghai Xuhui Central Hospital Zhongshan‐Xuhui Hospital, and the Shanghai Key Laboratory of Medical Epigenetics, the International Co‐laboratory of Medical Epigenetics and Metabolism (Ministry of Science and Technology) Institutes of Biomedical Sciences Fudan University Shanghai China

**Keywords:** 3D printing, anti‐counterfeiting label, bionic skin, cholesteric liquid crystal, drug delivery, hydroxypropyl cellulose, sensor, structural color

## Abstract

Hydroxypropyl cellulose (HPC), a cellulose derivative with biocompatibility, edibility, and exceptional solubility in many polar solvents, holds significant potential for biomedical applications. Within a specific concentration range, HPC undergoes self‐assembly to form cholesteric liquid crystals, which display distinct structural colors. These colors result from the interaction between incident light and the periodic nano‐architecture of HPC, providing long‐lasting visual effects that can be dynamically adjusted by factors such as concentration, temperature, and functional additives. This review includes the mechanisms underlying the genesis of structural colors and the regulation of HPCs while summarizing advanced techniques for fabricating HPC‐based materials with diverse configurations. Furthermore, through representative examples, we highlight the multifaceted applications of these materials in sensors, bionic skins, drug delivery, and anti‐counterfeiting labels. We also propose strategies to address current research and application challenges with the goal of exploring the potential of structural color HPCs for scientific breakthroughs and societal well‐being. We hope this review catalyzes HPC‐based structural color materials’ advancement and future biomedical applications.

## Graphical abstract

This review explores hydroxypropyl cellulose‐based structural coloration, focusing on its fundamental principles, fabrication techniques, and diverse applications, highlighting its potential for scientific and practical innovations.

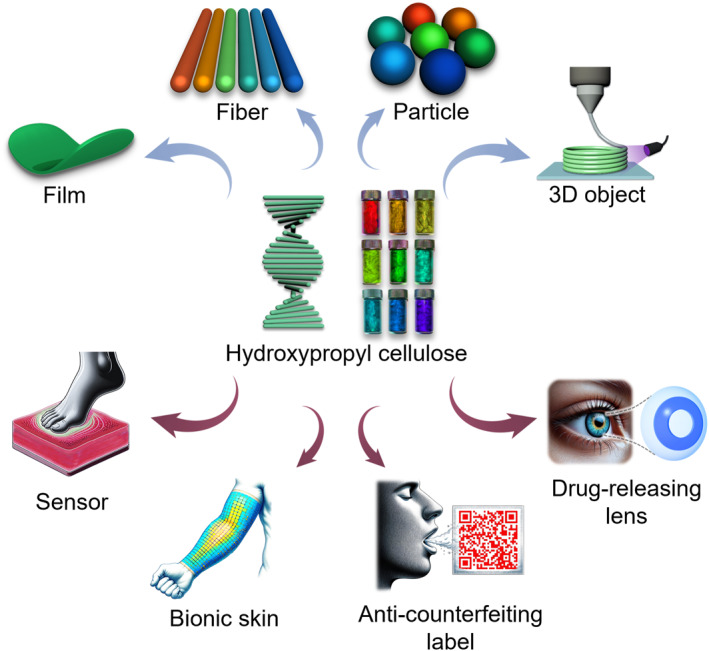

**Summary**

- Hydroxypropyl cellulose (HPC) self‐assembles into cholesteric liquid crystals, exhibiting unique structural colors that can be dynamically regulated by various factors, making it a promising material for diverse applications.
- This review summarizes the mechanisms of HPC‐based structural coloration, advanced fabrication techniques, and multifaceted applications, highlighting the potential of HPC for scientific advancements and biomedical applications.

## 1 Introduction

Structural coloration is a photonic phenomenon that arises from the interplay between light and the nanostructures of a substrate. This phenomenon is prevalent in diverse animals, insects, plants, and gemstones, with each entity’s unique structural color reflecting its distinct periodic structural features [1, 2, 3]. Inspired by this natural phenomenon, a variety of artificial structural color materials have been designed with spatially tailored periodic nanostructures arranged in one‐dimensional (1D), two‐dimensional (2D), or three‐dimensional (3D) configurations [4, 5, 6, 7, 8]. Unlike traditional dyes or pigments, these structural color materials offer fade‐resistant optical properties with tunable photonic bandgaps, driving innovations in optical technologies [9, 10, 11, 12, 13, 14, 15]. Generally, their fabrication techniques are categorized as top‐down or bottom‐up strategies [16, 17, 18]. Particularly, the bottom‐up strategy has gained significant interest due to its time and cost efficiency, ease of operation, and cross‐scale manufacturing capacities, enabled by self‐assembling basic units such as colloidal nanoparticles and block copolymers [19, 20, 21, 22, 23]. However, most of these methods involve intricate chemical reactions and raise biosafety concerns, limiting their applications in fields such as biomedicine [24, 25].

Over the past 2 decades, HPC, a semi‐synthetic long‐chain derivative, has emerged as a key component in the development of structural colors. Initial investigations into HPC‐based structural coloration can be traced back to the 1970s. However, recent studies have seen a significant increase due to the growing demand for its applications across various fields [26, 27, 28, 29, 30]. HPC is highly soluble in polar solvents such as water, ethanol, and dimethyl sulfoxide [31]. Within a specific concentration range in water, it self‐assembles into cholesteric liquid crystals (LCs), exhibiting lyotropic LC behavior. This arrangement reflects specific wavelengths of light based on the helical pitch [32, 33, 34, 35, 36, 37, 38]. Compared to other structural coloration methods, HPC‐based LCs offer unique responsive characteristics. Their visual color can be altered by factors such as humidity, temperature, metal ions, and chiral additives like glucose, making HPC a promising material for developing responsive photonic sensors [39, 40, 41, 42]. Furthermore, as a cellulose derivative, HPC benefits from cost‐efficiency, biocompatibility, and biodegradability due to its natural origins. These attributes make HPC‐based structural color materials outstanding candidates for biomedical applications [43, 44, 45].

In this review, we offer a comprehensive overview of advancements in HPC‐enabled structural coloration, with a focus on biomedical relevance. We begin by outlining the fundamental characteristics of HPC molecules. Subsequently, we explore the mechanisms driving structural coloration and stimuli‐responsive color changes. We then emphasize recent innovations in shaping structural colored HPCs into diverse forms, such as films, fibers, droplets/particles, and intricate 3D objects, while highlighting key features and fabrication techniques. Additionally, we discuss the promising applications of these HPC systems in areas like sensors, bionic skins, drug delivery, and anti‐counterfeiting labels. Lastly, we present an outlook on the future prospects of this field. Overall, we hope this review will catalyze the advancement of HPC‐based structural color materials and their future biomedical applications.

## 2 Characteristics of HPC‐Based Structural Coloration

### 2.1 Mechanism of Structural Color Production

**FIGURE 1:**
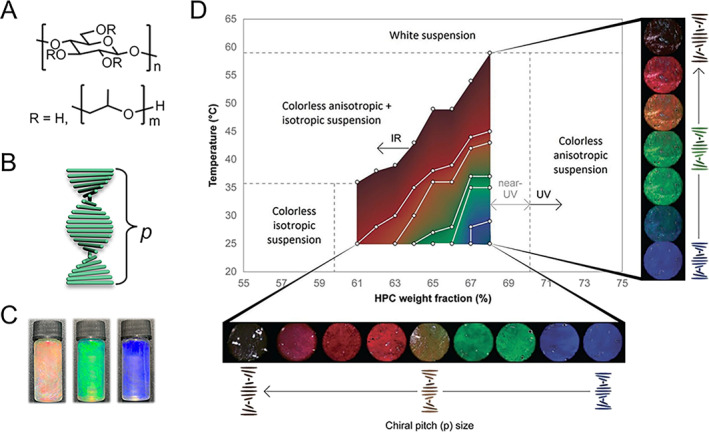
Mechanism of HPC‐based structural coloration. (A) Chemical structure of HPC molecules. (B) Schematic illustration of a CLC formed by HPC molecules and the helical pitch. (C) Structural color appearance of the HPC liquid crystals. Reproduced with permission [44]. Copyright 2022, Wiley‐VCH GmbH. (D) State diagram of an HPC aqueous solution. Reproduced under terms of the CC‐BY license [32]. Copyright 2023, The Authors, published by Wiley‐VCH GmbH. The optical images show the visual colors of an HPC aqueous solution with different HPC concentrations under different temperatures.

HPC is a widely available and biocompatible cellulose derivative (Figure 1A). At low concentrations, the HPC solution displays optical isotropy. However, once a critical concentration is exceeded, HPC molecules self‐assemble into a right‐handed chiral nematic phase, known as the cholesteric liquid crystal (CLC) [46]. These structures are characterized by continuously changing molecular orientation along a common direction (defined as the director) as shown in Figure 1B. When exposed to linearly polarized light perpendicularly, HPC CLCs preferentially reflect right circularly polarized (RCP) light while transmitting left circularly polarized (LCP) light unobstructed [47]. A concentration range of 60–70 wt% is crucial for aqueous HPC solutions to reflect RCP light across the visible spectrum, producing vibrant colors (Figure 1C) [48]. The helical pitch *p*, representing the axial distance for a complete 360° twist of the molecular director, determines the periodicity of the CLC mesophase. Generally, the wavelength *λ* of light reflected by the CLC system can be estimated using the De Vries equation [49]:

λ=npcosθ

where *n* refers to the average refractive index, and *θ* is the angle between the incident light direction inside the material and the helical axis. By adjusting these variables, the HPC system can display a broad spectrum of colors across the visible range. Furthermore, according to the De Vries equation, the displayed colors exhibit notable angle dependence relative to the observation direction [32]. As the observation angle increases, the color shifts visually from red to blue. Additionally, incorporating trace amounts of broadband light absorbers such as soluble black dye, carbon nanotubes (CNTs), or carbon black into HPC systems enhances color saturation without disrupting the CLC ordering of HPC molecules, resulting in a more striking visual display [50]. The hue, saturation, and value (HSV) color model and reflection spectrum are commonly used for the quantitative characterization of HPC colors [45, 51].

### 2.2 Mechanism of Structural Color Regulation

The HPC cholesteric mesophase exhibits highly uniform and wide‐range color tunability in thermodynamic equilibrium. Since it displays both lyotropic and thermotropic LC behaviors, its arrangement is influenced by solute concentration and ambient temperature. Specifically, as HPC concentration increases or temperature decreases, *p* reduces, leading to a corresponding shift in structural color, as illustrated in Figure 1D. Maintaining the hydration state is crucial for the HPC mesophase’s optical appearance and color response across the visible range, as its color display depends on a specific concentration range. Drying the HPC solution results in a decreased *p*, causing a blue shift in the reflective wavelength and ultimately rendering the sample colorless. To prolong the functional lifespan of the mesophase, encapsulation within media that prevents evaporation is beneficial. Common encapsulation materials include transparent glasses and flexible polymeric materials such as polydimethylsiloxane (PDMS) and polyethylene terephthalate (PET) [39, 40, 52].

Since the color of the HPC mesophase can be readily manipulated by altering *p*, mechanical compression or expansion can induce significant color changes—a phenomenon known as mechanochromism. Furthermore, HPC derivatives with variations in molecular weight, side chain length, or bonding species exhibit different twisting powers, providing an additional means to adjust *p* [53]. It has also been reported that additive salts can modulate the color of an HPC/water system, with the effect depending on concentration and salt component. This is likely due to the chaotropic effect, which alters the twisting angle and subsequently affects *p* [8, 54]. These multiple stimuli‐responsive properties position HPC as a versatile platform for biomimetic sensing and adaptive optical devices.

## 3 Tailoring HPC Structural Color Systems With Diverse Shapes

### 3.1 Films

**FIGURE 2:**
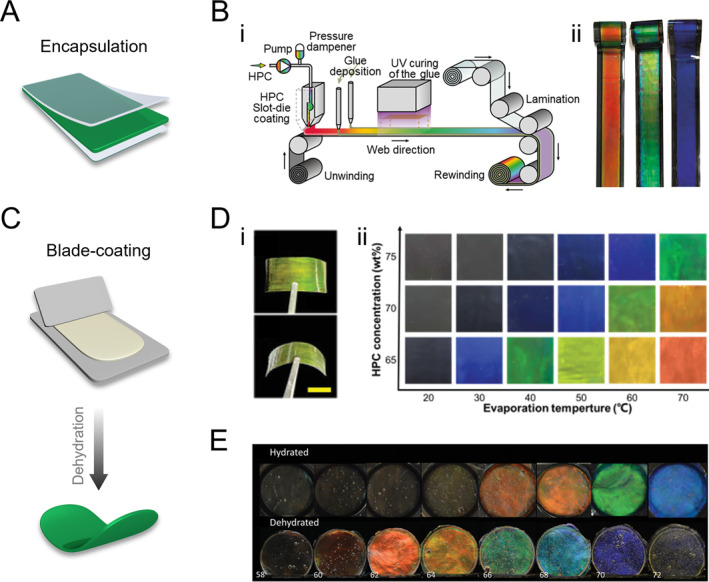
HPC structural color films. (A) Schematic illustration of the encapsulation strategy. (B) (i) Schematic illustration of the roll‐to‐roll mass production of PET‐encapsulated HPC photonic films. (ii) Optical images of HPC films with different colors. Reproduced under terms of the CC‐BY license [66]. Copyright 2018, The Authors, published by Springer Nature. (C) Schematic illustration of the blade‐coating strategy. (D) (i) Optical images of an HPC/acrylamide structural color solid film. Scale bar is 5 mm. (ii) Diagram of the color of the HPC/acrylamide films as a function of HPC concentration and the evaporation temperature during fabrication. Reproduced with permission [44]. Copyright 2022, Wiley‐VCH GmbH. (E) Optical images of HPC‐MA crosslinked hydrogel films (upper row) and their corresponding dehydrated state images (lower row) showing a blueshift. Reproduced under terms of the CC‐BY license [33]. Copyright 2022, The Authors, published by Wiley‐VCH GmbH.

Responsive polymer films exhibiting structural colors have garnered considerable interest in wearable biosensors due to their dynamic optical feedback and conformal adaptability [55, 56, 57, 58, 59, 60, 61, 62, 63, 64, 65]. Aqueous HPC solutions can form solid films upon water evaporation at room temperature, along with a blueshift of the reflection color. Encapsulation is an effective strategy employed to prevent evaporation and preserve the film’s color (Figure 2A). For large‐scale production, a roll‐to‐roll slot‐die coating, and lamination technique was used to continuously fabricate meter‐scale HPC structural color films (Figure 2B) [66]. Specifically, an HPC‐concentrated solution was coated onto a PET film substrate, followed by edge‐sealing and lamination for encapsulation. As the stock’s viscosity increased with HPC concentration, adjustments to the coating parameters were necessary to produce films of different colors. During fabrication, slight dehydration of the HPC solution and exposure to external forces led to color shifts and changes in molecular orientation. To mitigate these effects, an appropriate initial HPC concentration was selected, and a stress relaxation procedure was incorporated to achieve stable, desired colors.

To enhance the geometric adaptability of HPC mesophase and expand its application scope, various strategies have been devised to preserve visual colors in film‐shaped products. One effective approach involves immobilizing HPC CLC within a polymer hydrogel network. This is achieved by dissolving HPC in a pre‐gel of photo‐crosslinkable hydrogel at a sufficient concentration to induce CLC organization. The resulting mixture is then blade‐coated onto a substrate to form a thin film (Figure 2C). The monomers rapidly assemble into CLC structures to minimize shear deformation and are subsequently crosslinked via UV curing to stabilize the structure [43]. This composite hydrogel retains the inherent temperature and mechanical responsiveness of HPC while displaying color changes upon stimulation, displaying color changes upon stimulation (Figure 2Di) [44]. This suggests a delicate balance between *p*‐expansion induced by acrylamide and heating, and *p*‐contraction caused by water loss during drying, stabilizing the visible range of CLCs. The resulting colors in dry HPC films depend on initial concentration and drying temperature (Figure 2Dii). Another approach involves covalently crosslinking HPC to fix the CLC structure using difunctional crosslinkers such as glutaraldehyde (GA) [67, 68], or direct functionalization of HPC molecules with methacrylic anhydride (MA) [33]. Both HPC/GA and HPC‐MA systems retain their color after dehydration into dry films (Figure 2E). The HPC/GA system’s color retention is attributed to combined thermodynamic and kinetic arrest effects, while the HPC‐MA system’s color retention is due to the thermotropic expansion of *p* induced by the enthalpy of the polymerization reaction [33, 69].

### 3.2 Fibers

**FIGURE 3:**
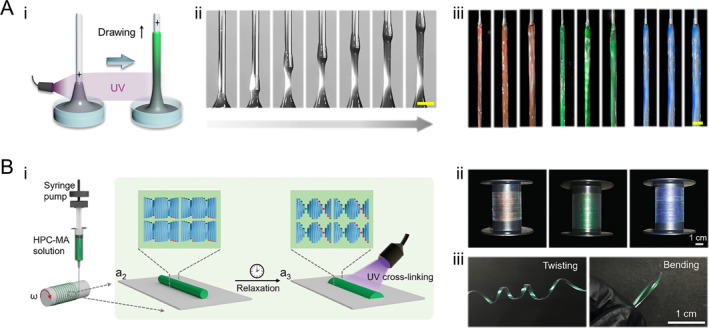
HPC structural color fibers. (A) (i) Schematic illustration and (ii) photographs of the processing of the HPC fibers by direct drawing and in situ UV‐polymerization. Scale bar is 3 mm. (iii) Optical image of the HPC fibers with various colors. Reproduced under terms of the CC‐BY license [77]. Copyright 2024, The Authors, published by American Association for the Advancement of Science. Scale bar is 5 mm. (B) (i) Schematic illustration of the fabrication process of HPC‐MA structural color fibers. (ii) Photographs of the HPC‐MA structural color fibers. (iii) Twisting and bending of the fibers. Reproduced under terms of the CC‐BY license [78]. Copyright 2024, The Authors, published by Wiley‐VCH GmbH.

HPC‐based photonic fibers are revolutionizing biomedical technologies through their inherent biocompatibility and dynamic optical signaling. While traditional polymer fibers can serve as textiles and optoelectronics, achieving multifunctional viable structural coloration requires engineering multi‐scale organization from molecular helical pitch to macroscopic fiber morphology [70, 71, 72, 73, 74, 75, 76]. Fluid coating and extrusion techniques have been widely adopted for the preparation of diverse polymer fiber types. However, achieving structural color fibers poses challenges due to the need for simultaneous manipulation of multiple length scales encompassing both basic unit organization and final fiber macromorphology. Recently, a direct drawing technology was proposed to enable the continuous generation of highly tunable multiscale properties in HPC fibers [77]. This technique involved employing a vertically moving nozzle that dipped into and drew a viscoelastic HPC pre‐gel solution. During the drawing process, stretching of the liquid interface occurred to form filaments which eventually pinched off. To prevent this undesired outcome, an in situ UV‐crosslinking setup was utilized where the continuously drawn filaments were irradiated at a fixed position above the liquid interface. The crosslinking reaction took place as the pre‐gel passed through this irradiated area, resulting in uniform diameter fibers without ductile failure (Figure 3Ai, Aii). Furthermore, this process retained CLC organization within the fiber structure, thereby imparting vivid structural coloration (Figure 3Aiii). Importantly, precise control over fiber diameter and structural color could be achieved by manipulating various processing parameters such as HPC pre‐gel formulation, nozzle dimensions and geometry, drawing speed, as well as UV irradiation position. In another research, HPC‐MA was utilized to continuously fabricate structural color fibers by combining extrusion, relaxation, and UV crosslinking [78]. HPC‐MA solutions were extruded through a nozzle and collected on a rotating mandrel, where the fibers underwent relaxation to re‐establish CLC orders. UV crosslinking then locked this structure in place, preserving the structural colors. By adjusting HPC‐MA concentration and humidity, the optical properties could be precisely controlled, achieving vibrant, tunable colors in meter‐long fibers (Figure 3B).

### 3.3 Droplets and Particles

**FIGURE 4:**
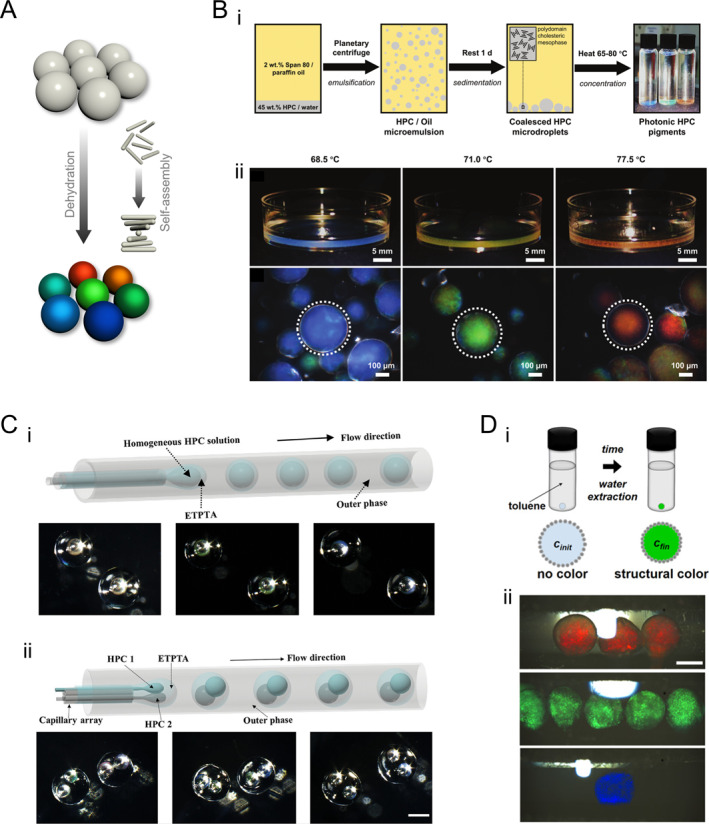
HPC structural color droplets and particles. (A) Schematic illustration of the HPC droplet/particle generation through dehydration. During this process, HPC molecules self‐assemble into CLC arrangements. (B) (i) Schematic illustration of the fabrication of HPC solid particles by heat‐drying droplets of HPC aqueous solution emulsified in paraffin oil. (ii) Photographs of different colored HPC particles generated at different heat‐drying temperatures. The upper panels are the photographs of the HPC particles in petri dishes and the lower panels are their corresponding microscopic images. Reproduced under terms of the CC‐BY license [85]. Copyright 2023, The Authors, published by Wiley‐VCH GmbH. (C) Schematic illustration of the fabrication process (upper part) and photographs (lower part) of the (i) single‐core and (ii) multi‐core HPC structural color microbubbles encapsulated within outer transparent shells. Reproduced under terms of the CC‐BY license [86]. Copyright 2024, The Authors, published by Wiley‐VCH GmbH. Scale bar is 100 μm. (D) (i) Schematic illustration of the generation of HPC‐based liquid marbles: a silica nanoparticle‐wrapped liquid marble embedding a low‐concentration HPC solution is immersed into toluene with a specific volume; a precise amount of water is extracted, forming CLCs and exhibiting colors. (ii) Liquid marbles with different colors generated by precise water extraction. Reproduced with permission [87]. Copyright 2020, The Authors, published by Wiley‐VCH GmbH. Scale bar is 1 mm.

Droplets serve as isolated compartments for biological and chemical reactions or as confined spaces to promote the self‐assembly of fundamental units such as colloids [79, 80, 81, 82, 83, 84]. HPC droplets can be generated through emulsification in oil, enabling the fabrication of HPC structural color microparticles via a heating‐drying process. This process balances thermally‐induced red‐shift and evaporation‐induced blue‐shift with increasing HPC concentration, leading to HPC self‐assembly into CLCs (Figure 4A) [85]. Specifically, 45 wt% aqueous HPC solution was emulsified into HPC‐in‐oil droplets using planetary centrifugal mixing (Figure 4Bi). This resulted in a CLC mesophase with low viscosity, facilitating easy processing. After allowing the CLC structure to stabilize, solid HPC particles with structural color were obtained through heating and drying at 65°C–80°C. The particle color can be adjusted by controlling the heating and drying temperature (Figure 4Bii). Moreover, HPC self‐assembly can be precisely controlled within a confined space. By utilizing droplet microfluidics and a simple solvent extraction process, HPC molecules were concentrated and self‐assembled into CLC phases, forming microbubbles with transparent shells that exhibit structural colors (Figure 4Ci) [86]. By adjusting the microfluidic flow rates and HPC concentrations, a variety of color combinations can be achieved in these microbubbles (Figure 4Cii). Additionally, these HPC microbubbles retain their thermos‐responsiveness, suggesting potential applications in underwater optical sensors, anti‐counterfeiting measures, and biosensing technologies.

Liquid marbles, or sessile drops coated with particles, have also been exploited by controlling the self‐assembly of HPCs [87]. In this process, a low‐concentration HPC solution is first formed into droplets within a two‐phase system. These droplets are then rolled on a bed of fumed silica nanoparticles to create liquid marbles that encapsulate HPC in the biphasic regime. Subsequently, the liquid marbles were immersed in toluene to extract a specific amount of water, inducing HPC to form a CLC structure that selectively reflected color (Figure 4Di). The color of the resulting liquid marbles can be finely tuned by adjusting the volume of toluene used (Figure 4Dii). These HPC liquid marbles maintain their inherent responsiveness to temperature and mechanical stimuli, reporting changes through variations in color. Additionally, their sensitivity to HPC concentration makes them useful for monitoring various solvents that can dissolve HPC as changes in solvent concentration are reflected in changes in color.

### 3.4 Complex 3D Objects

The inherent shear‐thinning rheology of HPC CLCs enables their use as functional bioinks for direct ink writing, an additive manufacturing technique that has seen rapid advancements recently and offers great potential for creating customized 3D architectures [88, 89, 90, 91, 92, 93, 94]. The printability of HPC‐based inks relies on their rheological properties. Aqueous solutions of HPC exhibit shear thinning behavior. When extruded from a nozzle, their viscosity was reduced due to applied shear, forming filaments. However, despite restoring their intrinsic viscosity after printing, these filaments fail to retain their shape due to the fluidic nature of HPC. To address this, supramolecular gelling agents like gelatin, polyethylene glycol (PEG), or cellulose nanofibrils can be incorporated into HPC solutions, resulting in physically crosslinked viscoelastic gels [45, 50, 95, 96]. These composite materials maintain the shear‐thinning behavior of HPC, flowing easily under high shear rates but transitioning into a self‐supporting gel state at lower shear rates. This makes them ideal for advanced ink processing techniques. With computerized control, these HPC inks can be precisely extruded from nozzles, enabling the creation of complex patterns over large areas. Notably, PEG not only modifies rheology but also influences *p* during post‐printing drying. By fine‐tuning PEG concentration and drying temperature, photonic solids with a wide range of reflected colors across the visible spectrum can be produced through kinetic trapping of *p* [95].

**FIGURE 5:**
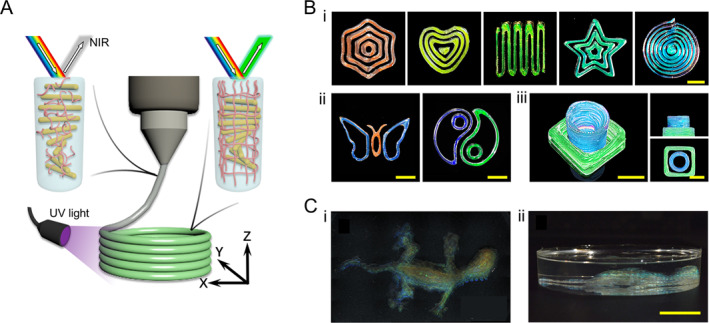
HPC‐based photonic inks and 3D‐printed complex objects. (A) Schematic illustration of the 3D printing of a UV‐curable HPC ink. The ink flows through the nozzle and forms filaments on a substrate layer by layer. A UV light source is used to polymerize the ink and fix its structure. Reproduced with permission [45]. Copyright 2022, The Authors, published by National Academy of Sciences. (B) (i and ii) 2D patterns and (iii) 3D objects printed by an HPC/gelatin ink. The visual color can be controlled by the HPC content. Reproduced with permission [45]. Copyright 2022, The Authors, published by National Academy of Sciences. Scale bars are 4 mm. (C) Photos of a chameleon‐shaped 3D object printed by an MA‐functionalized HPC ink viewed from (i) top and (ii) side view. Reproduced under terms of the CC‐BY license [33]. Copyright 2022, The Authors, published by Wiley‐VCH GmbH. Scale bar is 1 cm.

In addition to physical crosslinking, several chemical crosslinking strategies have been explored to enhance the versatility of HPC‐based structural color materials. Zhang et al. introduced a real‐time solidification method that synergizes with the printing process [45]. Their refined ink formulation, consisting of HPC, gelatin, and a UV‐polymerizable pre‐gel, utilizes in situ UV irradiation to form a chemically crosslinked network, effectively maintaining the shape of printed filaments (Figure 5A). This approach enables the layer‐by‐layer fabrication of complex 3D structural color objects (Figure 5B) [45]. Alternatively, adding a GA crosslinker directly to HPC aqueous solutions allows thermal‐induced crosslinking of extruded filaments [32]. However, the relatively long crosslinking time of this method limits its application in constructing complex 3D objects. To overcome this, HPC molecules can be functionalized with methacrylic anhydride (MA), enabling immediate in situ UV‐curing of the HPC‐MA ink after extrusion. This results in structurally stable filaments (Figure 5C) [33].

## 4 Applications of HPC Structural Color Systems

### 4.1 Sensors

**FIGURE 6:**
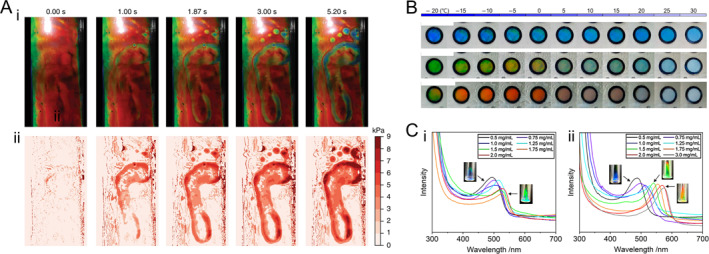
HPC‐based structural color sensors. (A) (i) A series of optical images captured frame‐by‐frame recording the footprints left by a participant on an encapsulated red HPC film. (ii) False‐color pressure maps plotted according to the optical signals captured by a camera from the corresponding images in (i). Reproduced under terms of the CC‐BY license [66]. Copyright 2018, The Authors, published by Springer Nature. (B) Photographs showing the color variation of an encapsulated ethylene glycol‐doped HPC temperature sensor with different ethylene glycol concentrations at different temperatures. 60 wt% HPC is dissolved in the mixed solvent, and the ethylene glycol content in the mixed solvent is (i) 30 wt%, (ii) 20 wt%, and (iii) 10 wt%, respectively. Reproduced with permission [51]. Copyright 2022, MDPI. (C) Reflection spectra of (i) 64 wt% HPC aqueous solution and (ii) 70 wt% PBA‐functionalized HPC aqueous solution containing glucose of different concentrations. The inserted photographs show the visual colors of several representative samples. Reproduced with permission [105]. Copyright 2022, Wiley Periodicals LLC.

Colorimetric sensors, which provide intuitive feedback through visually detectable color changes in response to stimuli, have garnered considerable attention in real‐time physiological monitoring [97, 98, 99, 100, 101, 102, 103, 104]. HPC, with its inherent mechanical and temperature responsiveness, is particularly advantageous for this purpose. By encapsulating HPC mesophase within flexible films, an HPC colorimetric sensor can capture real‐time pressure distributions through mechanical color alterations. For example, this method can spatially record forces exerted by human footprints, as illustrated in Figure 6Ai [66]. The resulting optical feedback can be captured and quantified using a smartphone camera, producing a time‐varying 2D pressure map (Figure 6Aii). To monitor food freshness, an edible shellac‐encapsulated evaporative discoloration timer was developed, leveraging HPC’s humidity responsiveness [106]. For temperature sensing applications where water freezing below 0°C is a concern, the incorporation of ethylene glycol as a coolant modifies hydrogen bond formation within the HPC system, lowering the freezing point to below −20°C (Figure 6B) [51]. Furthermore, HPC aqueous solution can serve as a glucose sensor due to the chiral centers in glucose molecules, which increase polarization with concentration (Figure 6C) [105]. Enhancing this capability, phenylboronic acid (PBA)‐functionalized HPC within the CLC mesophase exhibits improved glucose response. PBA side chains form covalent complexes with glucose, leading to increased polarization due to steric and electrostatic repulsion between HPC molecules arising from augmented side chain bulkiness and charge density. This results in heightened sensitivity for colorimetric glucose detection.

### 4.2 Bionic Skins

**FIGURE 7:**
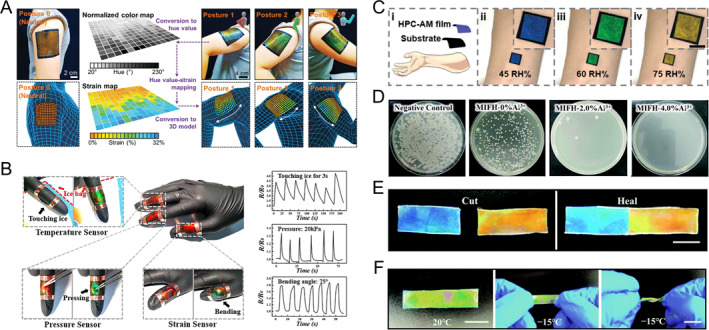
HPC‐based structural color bionic skins. (A) A PDMS‐packaged HPC mesophase serving as wearable film sensors attached to a human arm, monitoring mechanical signals of human motions. The spatial photonic responses (upper row) are imaged by a camera and processed into a real‐time 3D stimuli map (lower row). Reproduced with permission [40]. Copyright 2019, Wiley‐VCH GmbH. (B) A dual‐signal sensing electric skin that reports stimuli via visual color variation and electrical feedback. Reproduced with permission [43]. Copyright 2020, National Academy of Sciences. The stimuli of environmental temperature, pressure, and strain can cause changes in the relative electric resistance of the device, providing precise responses. (C) (i) Schematic illustration and (ii–iv) optical images of the bionic skin fabricated by an HPC film sensor, which could report environmental humidity by color changes. Reproduced with permission [44]. Copyright 2022, Wiley‐VCH GmbH. (D) Photographs of the *E. coli* bacteria colonies on agar plates after contacting with the Al^3+^‐containing bionic skins with different Al^3+^ concentrations. Reproduced with permission [41]. Copyright 2021, Royal Society of Chemistry. The survival rate of bacteria decreases with an increase in the Al^3+^ concentration. (E) Self‐healing ability of the bionic skin. Reproduced with permission [41]. Copyright 2021, Royal Society of Chemistry. Two pieces of cut fragments are attached together into one piece owing to the coordination bond and the hydrogen bond. (F) The anti‐freezing ability of the bionic skin. Reproduced with permission [41]. Copyright 2021, Royal Society of Chemistry. The visual color display and flexibility are maintained at −15°C.

Bionic skin, a flexible wearable device that mimics biological skin functions, holds significant research value in smart robotics and medicine [107, 108, 109, 110, 111, 112, 113, 114]. HPC‐based structural color systems, with their multi‐stimuli responsiveness, can serve as visual feedback interfaces. Jeong et al. developed a PDMS‐packaged HPC film device featuring a pixelated architecture to monitor human motions in real‐time via visual color signals [40]. When adhered to the human body, the strain from motions alters *p*. The pixelated design enables region‐specific color mapping, which can be captured and analyzed by cameras or other instruments for large‐area motion tracking (Figure 7A). Furthermore, an HPC‐based electronic skin was created by incorporating conductive additives. This skin provides both optical and electrical feedback for detecting human motion, enhancing its sensing capabilities (Figure 7B) [43].

In addition to mechanical perception, biological skin senses environmental conditions and offers antibacterial, self‐healing, and cold‐resistant properties. To mimic these features, HPC‐based structural color films have been devised to monitor temperature and detect humidity based on the corresponding responsiveness of CLC (Figure 7B, C) [43, 44]. For broader applicability, an HPC CLC system integrated with Al^3+^ was developed as a versatile multitasking bionic skin [41]. Notably, Al^3+^ exhibited potent antimicrobial activity against both gram‐positive and gram‐negative bacteria (Figure 7D). Notably, Al^3+^ exhibited potent antimicrobial activity against both gram‐positive and gram‐negative bacteria (Figure 7E). This self‐healing capability allowed the device to resist deformations while restoring its conductivity and other functions. Furthermore, Al^3+^ acted as a physiological salt, lowering the freezing point of the HPC‐aqueous system and enabling operation at −15°C in extremely cold environments (Figure 7F). Overall, the integration of multiple functions into HPC structural color bionic skins not only mimics but also surpasses certain aspects of biological skin. This breakthrough opens up new avenues for biomimetic research and medical device development.

### 4.3 Drug Delivery

**FIGURE 8:**
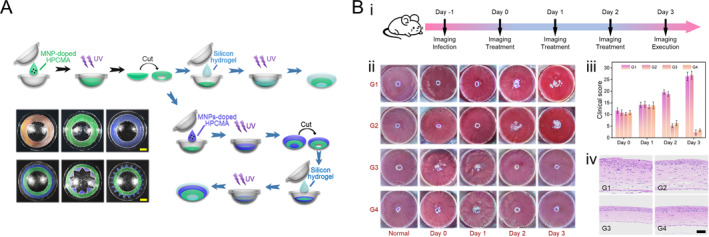
HPC‐based structural color drug delivery contact lens. Reproduced with permission [115]. Copyright 2024, Wiley‐VCH GmbH. (A) Schematic illustration of the fabrication process of the SCCLs and their photographs. (B) In vivo evaluation of AMP‐loaded SCCLs conducted using an animal model. (i) Experimental flow chart. (ii) Corneal and anterior chamber images of *E. coli*‐infected mice from day 0 to day 3. (iii) Clinical scoring of corneal condition across 4 days for different treatment groups. (iv) H&E‐stained corneal sections of mice on day 3. Scale bar is 50 μm.

The excellent biocompatibility of HPC allows it to be used in the biomedical field. Notably, with the development of HPC‐MA hydrogels, HPC‐based liquid crystals can maintain structural stability and exhibit color in aqueous environments. Besides, HPC‐MA, which can crosslink into a polymer hydrogel network, also serves as a carrier for drug release, laying the foundation for in vivo studies. In a recent study, a structural color contact lens (SCCL) based on CLCs from HPC‐MATo was developed for aesthetic and therapeutic applications [115]. These SCCLs, created through self‐assembly and UV crosslinking, provide vibrant, chemical‐free color without pigment contamination (Figure 8A). By incorporating antimicrobial peptides (AMPs) such as polymyxin B and vancomycin, the SCCLs demonstrated antibacterial efficacy against both Gram‐negative and Gram‐positive bacteria. In a bacterial keratitis mouse model, AMP‐loaded SCCLs showed therapeutic effects comparable to commercial levofloxacin eye drops (Figure 8B). The treated groups exhibited significant reductions in inflammation, *Escherichia coli* levels, and clinical scores, with histopathological analysis revealing preserved corneal structure and reduced inflammatory cell infiltration. These findings suggest that AMP‐loaded SCCLs could provide both cosmetic and therapeutic advantages, particularly in preventing and treating bacterial keratitis. This study also provides novel insights into the application of HPC CLCs in vivo experiments.

### 4.4 Anti‐Counterfeiting Labels

**FIGURE 9:**
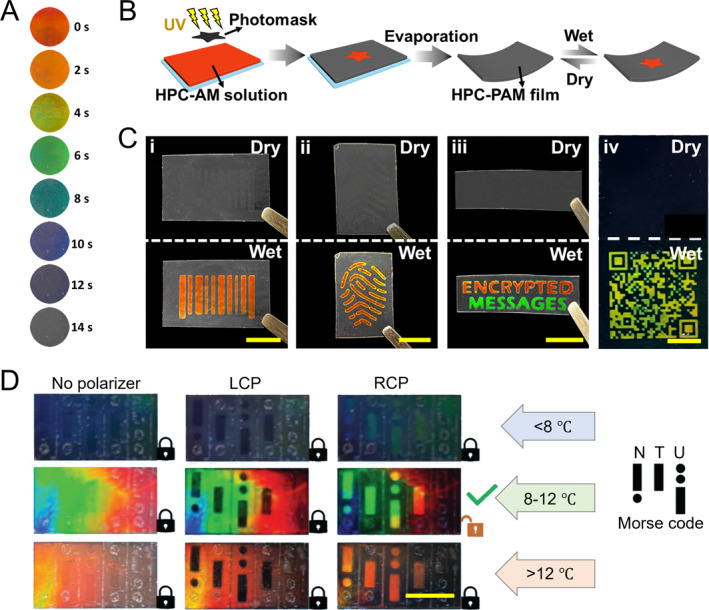
HPC‐based structural color anti‐counterfeiting labels. (A) Color variation of the HPC‐PAM hydrogel during the photo‐crosslinking of a UV light source. The color blue shifted and finally vanished with the increase in the crosslinking degree. Reproduced with permission [44]. Copyright 2022, Wiley‐VCH GmbH. (B) Schematic illustration of the fabrication of a humidity‐triggered HPC‐PAM anti‐counterfeiting label. Reproduced with permission [44]. Copyright 2022, Wiley‐VCH GmbH. (C) Anti‐counterfeiting labels encrypting patterns of (i) bar code, (ii) fingerprint, (iii) text, and (iv) QR code. The patterns were invisible during the dry state and appeared in the wet state. Reproduced with permission [44]. Copyright 2022, Wiley‐VCH GmbH. Scale bars are 0.2 mm. (D) Decoding of a Morse code encrypted HPC‐based label through dual‐decryption of polarization and temperature. Reproduced with permission [123]. Copyright 2023, Wiley‐VCH GmbH. Scale bar is 1 cm.

Photonic systems, particularly structural color systems, offer significant applications in anti‐counterfeiting technology due to their stability and fade resistance [116, 117, 118, 119, 120, 121, 122]. Recently, HPC‐based structural colors have attracted attention for their simple preparation and environmental friendliness [44, 54, 123, 124]. For instance, Zhang et al. developed a series of security labels utilizing the humidity responsiveness of HPC [44]. They created a composite HPC‐polyacrylamide (PAM) hydrogel with an interpenetration network by photo‐crosslinking an aqueous mixture of HPC and acrylamide. Adjusting the UV‐crosslinking time modified the material’s color (Figure 9A). During polymerization, compression within the CLC framework occurred, leading to a blue shift in color as UV irradiation time increased. Excessive cross‐linking disrupted CLC ordering, causing permanent fading. By using a pre‐designed photomask, specific areas of the HPC pre‐gel mixture with CLC ordering were selectively exposed to UV light. This allowed colors to disappear in exposed regions while masked areas remained unchanged, enabling customizable structural color patterns with precise multi‐color control through cross‐linking times. Importantly, non‐exposed areas maintained humidity responsiveness, supporting the design of HPC‐based anti‐counterfeiting labels (Figure 9B). The non‐exposed areas exhibited reversible fading and color‐revealing reactions in dry and humid environments, as shown in Figure 9C. Using corresponding photomasks, information such as barcodes, fingerprints, texts, and QR codes can be encrypted. When exposed to water vapor, like from human respiration, the concealed colored patterns are activated and revealed. Smartphones can easily recognize these patterns, providing a convenient, cost‐effective, and intuitive solution for information encryption and anti‐counterfeiting. In another instance, HPC CLCs were integrated into a crosslinked poly(N‐isopropyl acrylamide) (PNIPAM) network to enable a multilevel information encryption system [123]. This system utilizes UV exposure duration to finely tune the crosslinking density and temperature‐responsive range, increasing the complexity of information storage. Additionally, the polarization states of reflected light can be adjusted with a waveplate, adding another layer to the encryption/decryption process for both LCP and RCP light (Figure 9D).

### 4.5 Others

In addition to the aforementioned applications mainly focused on HPC films, there is a growing exploration of the practical uses of HPC structural color systems across various fields. The high biocompatibility and edibility of HPC have ignited research in food decoration and cosmetics, leading to the creation of 3D structural color objects that combine gelatin and HPC, as well as particles made entirely of HPC [50, 85]. By synergistically incorporating temperature‐sensitive hydrogels, HPC structural color fibers can provide real‐time visual indications of environmental temperature changes. This makes HPC a highly promising material for next‐generation smart textiles, given the growing demand for optical display functionality in this area [78, 125]. Moreover, the degradability of both HPC and crosslinked HPC‐MA makes them attractive candidates for implantable devices, such as smart drug delivery systems and sensors [111, 112]. By combining HPC with other functional ingredients, complex and customizable structural color systems can be developed for a wide range of everyday and industrial applications in the future.

## 5 Summary and Outlook

In recent years, HPC has emerged as a promising material for structural coloration via self‐assembly. Its low cost, wide availability, good solubility, biocompatibility, and edibility make it highly appealing compared to synthetic polymers or colloids. Additionally, the inherent multi‐responsive nature of HPC‐based CLC mesophase offers extensive opportunities for exploring its photonic properties and various applications. This review summarizes the fundamental principles, technical advancements, and practical applications of HPC‐based structural coloration. We start by explaining the origins and regulatory mechanisms of HPC structural coloration. Next, we introduce various forms of HPC CLC systems, including films, fibers, droplets/particles, and complex 3D objects, along with the different processing technologies used to create them. Finally, we focus on the biomedical applications of structurally colored HPCs, including biosensors, bionic skins, and stimuli‐responsive drug delivery systems, while briefly acknowledging their potential in other fields such as anti‐counterfeiting labels.

The investigation of HPC‐based structural coloration is gaining traction, while challenges persist across various aspects. While the reflective wavelength of HPC CLC mesophases can be predicted by the De Vries equation, the visual appearance of HPC‐based structural colors also depends on factors such as the alignment and arrangement of internal CLC domains and the viewing angle [69]. During the processing of structural colored HPCs, dynamic changes in CLC domains are influenced by crosslinkers, evaporation kinetics, and shear forces. Understanding these mechanisms is crucial for gaining insights into the optical properties of self‐assembled HPCs, including color purity, intensity, polarization, and scattering. Moreover, compared to other types of structural coloration, many potential applications of HPC structural color systems remain unexplored. Future studies should prioritize elucidating HPC's dynamic interactions with cells and tissues to advance its use in implantable biointerfaces and regenerative medicine. Additionally, technologies for processing structural colored HPC are primarily at the laboratory stage, with a significant gap between proof‐of‐concept studies and practical industrial applications. To bridge this gap, precise manipulation at the molecular level, combined with simultaneous control over multiscale structuring, is essential. Besides, to accelerate clinical translation, scalable manufacturing methods that preserve HPC's photonic functionality under sterilization conditions must be developed. In conclusion, with its emerging prominence in the field of self‐assembly and structural coloration, HPC holds the potential to catalyze numerous innovative breakthroughs, paving the way for its widespread applications.

## Author Contributions

Luoran Shang and Zhuohao Zhang conceived the idea. Zhuohao Zhang and Luoran Shang wrote the original manuscript. Luoran Shang revised the manuscript.

## Conflicts of Interest

Luoran Shang is an executive editor for *Smart Medicine* and was not involved in the editorial review or the decision to publish this article. All authors declare that there are no competing interests.
